# Phytochemicals in Cancer Immune Checkpoint Inhibitor Therapy

**DOI:** 10.3390/biom11081107

**Published:** 2021-07-27

**Authors:** Juwon Lee, Youngjin Han, Wenyu Wang, HyunA Jo, Heeyeon Kim, Soochi Kim, Kyung-Min Yang, Seong-Jin Kim, Danny N. Dhanasekaran, Yong Sang Song

**Affiliations:** 1 Cancer Research Institute, College of Medicine, Seoul National University, Seoul 03080, Korea; isone227@snu.ac.kr (J.L.); youngjin.han@snu.ac.kr (Y.H.); wywang@snu.ac.kr (W.W.); whgusdk25@snu.ac.kr (H.J.); heekim312@snu.ac.kr (H.K.); 2 WCU Biomodulation, Department of Agricultural Biotechnology, Seoul National University, Seoul 08826, Korea; 3 SK Biopharmaceuticals Co., Ltd., Seongnam-si 13494, Korea; 4 Interdisciplinary Program in Cancer Biology, Seoul National University, Seoul 03080, Korea; 5 Department of Neurology and Neurological Sciences, School of Medicine, Stanford University, Stanford, CA 94304, USA; skim245@stanford.edu; 6 MedPacto Inc., 92, Myeongdal-ro, Seocho-gu, Seoul 06668, Korea; yangkm@snu.ac.kr (K.-M.Y.); jasonsjkim@snu.ac.kr (S.-J.K.); 7 Precision Medicine Research Center, Advanced Institute of Convergence Technology, Seoul National University, Suwon 16229, Korea; 8 Department of Transdisciplinary Studies, Graduate School of Convergence Science and Technology, Seoul National University, Suwon 16229, Korea; 9 Department of Cell Biology, The University of Oklahoma Health Sciences Center, Oklahoma City, OK 73104, USA; danny-dhanasekaran@ouhsc.edu; 10 Stephenson Cancer Center, The University of Oklahoma Health Sciences Center, Oklahoma City, OK 73104, USA; 11 Department of Obstetrics and Gynecology, Seoul National University College of Medicine, Seoul 03080, Korea

**Keywords:** phytochemical, immune checkpoint, PD-1, PD-L1, cancer immunotherapy

## Abstract

The interaction of immune checkpoint molecules in the tumor microenvironment reduces the anti-tumor immune response by suppressing the recognition of T cells to tumor cells. Immune checkpoint inhibitor (ICI) therapy is emerging as a promising therapeutic option for cancer treatment. However, modulating the immune system with ICIs still faces obstacles with severe immunogenic side effects and a lack of response against many cancer types. Plant-derived natural compounds offer regulation on various signaling cascades and have been applied for the treatment of multiple diseases, including cancer. Accumulated evidence provides the possibility of efficacy of phytochemicals in combinational with other therapeutic agents of ICIs, effectively modulating immune checkpoint-related signaling molecules. Recently, several phytochemicals have been reported to show the modulatory effects of immune checkpoints in various cancers in in vivo or in vitro models. This review summarizes druggable immune checkpoints and their regulatory factors. In addition, phytochemicals that are capable of suppressing PD-1/PD-L1 binding, the best-studied target of ICI therapy, were comprehensively summarized and classified according to chemical structure subgroups. It may help extend further research on phytochemicals as candidates of combinational adjuvants. Future clinical trials may validate the synergetic effects of preclinically investigated phytochemicals with ICI therapy.

## 1. Introduction

Phytochemicals are bioactive compounds that are naturally produced in plants such as fruits and vegetables. Phytochemicals are essential for plant growth and maintenance by functioning as secondary metabolites, protecting them from animals, insects, and microorganisms, or coloring plants as pigments [1]. Traditional use and continuous observation have found that the extracts of certain plants have therapeutic and preventive effects on human diseases. This is defined as phytotherapy, which a medical science that uses plant extracts or phytochemicals to treat diseases or improve health. The natural compounds contained in plant extracts display a wide range of biological activities in the human body, such as antioxidation, immune-boosting, anti-inflammation, and maintenance of cardiovascular health [2]. Accumulative studies have reported epidemiological, laboratory, and clinical evidence supporting the tumor-suppressive effects of phytochemicals [3]. The tumor-suppressive mechanisms of phytochemicals include disrupting redox balance of cancer cells, inhibiting proliferation, inducing cell cycle arrest, and apoptosis and boosting anti-cancer immunity. These biomodulatory effects of phytochemicals lead to decreased cancer cell growth, progression, and chemotherapy resistance.

Currently, there has been an increasing interest in cancer immunotherapy, which artificially stimulates the anti-cancer immune system to eliminate malignancy. To restore the suppressed ability of immune cells to recognize cancer cells, monoclonal antibodies (mAbs) have been adopted as immune checkpoint inhibitors (ICIs) by blocking the interactions of immune checkpoints between cancer and immune cells. The idea of fighting against cancer by “reactivating” cancer-inactivated natural immunity in patients was captivating in that the survival rate of patients with advanced-stage and metastatic cancers has been improved dramatically with current clinical practices of anti-cancer immunotherapy [4,5,6]. Ipilimumab, an anti-CTLA-4 mAb, was approved as the first ICI by the Food and Drug Administration (FDA) in 2011 [7]. Since then, a total of seven types of ICIs targeting CTLA-4 and PD-1/PD-L1 have been approved for cancer immunotherapy in the last decade. The introduction of pembrolizumab and nivolumab, anti-PD-1 mAbs, has remarkably advanced cancer immunotherapy in treating many cancer types, which include metastatic melanoma, non-small-cell lung cancer (NSCLC), head and neck squamous cell carcinoma, and Hodgkin’s lymphoma [8]. However, there are limitations in the clinical use of ICIs in cancer treatment. Due to an unstable immune system, many patients receiving immunotherapy experience immune-related adverse events (irAE) that involve gastrointestinal toxicity, endocrine toxicity, and dermatologic toxicity [9]. Furthermore, the cost of treatment is enormously expensive in that it may cost more than $100,000 per patient during the course of treatment, depending on the type of immunotherapy and the number of cycles administered to the patient [10,11]. Although ICI has demonstrated high response rates in some cancer types, the majority of cancer patients still do not respond to ICI immunotherapy, and response rates for certain types of cancer, including ovarian cancer, are less than 5% [12,13].

ICIs are now used as single agents or in combination with other therapeutic agents as a first- or second-line treatment for several cancer types, including metastatic melanoma and lung cancer [14]. However, using multiple drugs may yield severe side effects and seriously deteriorate a patient’s quality of life. Moreover, using multiple drugs increases the cost of treatment and patient burden. There are urgent needs for reducing side effects and medical expenses while enhancing the efficacy of the treatment.

**Figure 1:**
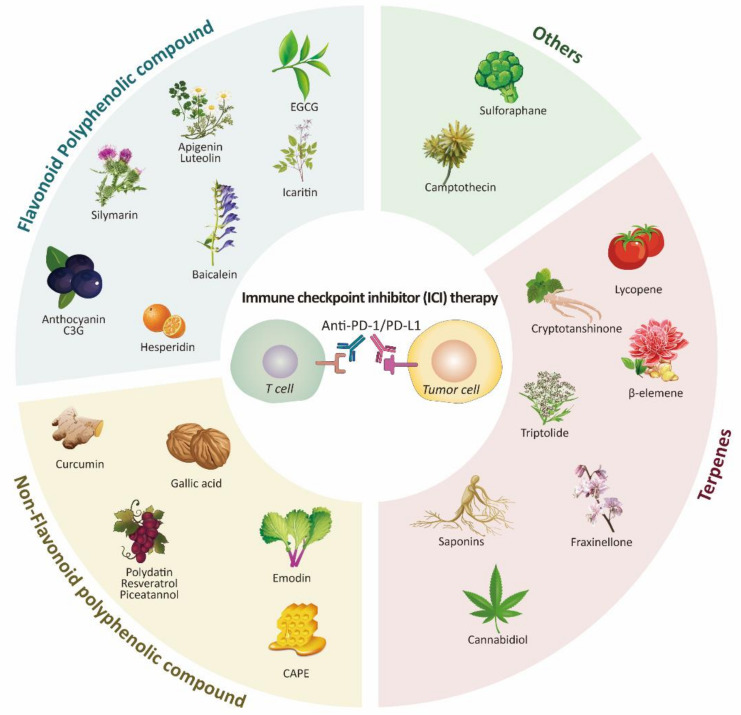
Various phytochemicals and their representative sources may modulate anti-PD-1/PD-L1 ICI therapy. Isolated phytochemicals and their sources are shown according to the structural subgroups. Flavonoid polyphenolic compounds include EGCG, icaritin, apigenin, luteolin, baicalein, silymarin, anthocyanin, C3G, and hesperidin. Non-flavonoid polyphenolic compounds include curcumin, gallic acid, polydatin, resveratrol, piceatannol, emodin, and CAPE. Terpenes include lycopene, cryptotanshinone, β-elemene, triptolide, fraxinellone, saponins, and cannabidiol. Others include sulforaphane and camptothecin. EGCG: epigallocatechin gallate; C3G: Cyanidin 3-*O*-glucoside; CAPE: caffeic acid phenethyl ester.

A growing body of evidence has shown the efficient modulation of the tumor microenvironment (TME) by phytochemicals. Accumulative studies suggest that the effect of cancer immunotherapy may be boosted with the combination of certain phytochemicals, such as resveratrol and curcumin [15]. In this review, we introduce phytochemicals shown to regulate immune checkpoints or affect ICI therapy and suggest them as potential candidates for the combination partner of ICI therapy to provide insight into immune checkpoint modulation and inspiration for future study (Figure 1).

## 2. Molecular Machineries Regulating PD-1/PD-L1 in the Tumor Microenvironments

Our body has a defensive immune system that protects us from external intruders (e.g., viruses and toxins) and harmful internal abnormalities (e.g., tumorigenesis). Unlike normal cells, cancer cells have specific antigens on their surface and are eliminated by the immune system in the early stages. However, as the tumor grows aggressively, clones of cancer cells may evade immune surveillance by disturbing the balance of stimulatory and inhibitory signaling of immune cells via B7-CD28 family interaction. Interactions between ligands and the B7-CD28 family receptors are called “immune checkpoints” and play essential roles in T cell co-stimulation and co-inhibition, maintaining immune homeostasis [16]. In the TME, cancer cells escape from immune cells through the expression of co-inhibitory B7 family ligands such as programmed death-ligand 1 (PD-L1). Their binding to the T cell co-inhibitory CD28 family receptors, including programmed cell death receptor 1 (PD-1) and cytotoxic T-lymphocyte-associated protein 4 (CTLA-4), results in decreased immune cell activity, allowing cancer cells to escape from immune surveillance [17].

### 2.1. PD-1

PD-1, also known as CD279, is an inhibitor of both adaptive and innate immune responses. PD-1 is expressed on activated T cells, NK cells, B cells, macrophages, and dendritic cells (DCs), but are most highly expressed on tumor-specific T cells in TME [18,19]. The activities of PD-1 and its ligand PD-L1 are responsible for reducing activation, proliferation, and cytokine secretion of T cells in TME, resulting in decreased anti-tumor immune responses [20]. PD-1 transcription may be triggered by transcription factors including nuclear factor of activated T cells (NFAT), NOTCH, Forkhead box O1 (FOXO1), and IFN regulatory factor 9 (IRF9) [21]. The two conserved upstream regulatory regions B and C (CR-B and CR-C) are important for the expression of PDCD-1 (PD-1 gene), containing multiple potential binding sites for transcription factors. CR-B and CR-C are heavily methylated in naive T cells, but after the first antigen encounter, both regions are demethylated, coinciding with PD-1 expression [21]. NFAT transcription factors have been proposed as effective modulators of this effector versus a hyporesponsiveness T-cell state [22]. NFATs control effector genes and T-cell functions by forming a protein complex with AP-1 (c-Fos and Jun proteins) in effector T cells, which is caused by effective co-stimulation signaling. Meanwhile, monomeric NFATs are present at a high level in exhausted T cells. The formation of NFAT1 monomers activates transcription of genes related to T cell dysfunction [23]. Moreover, binding to the promoter of the PD-1 gene by IFN-α and IRF9 in exhausted T cells can result in PD-1 expression [24]. The PD-1 promoter, which is demethylated during chronic infections, results in the increased expression in exhausted CD8+ T cells. FOXO1 transcription factor binds to the PD-1 promoter, enhancing its expression [25]. Furthermore, TGF-β, IL-2, IL-21, IL-15, IL-7, and Type 1 IFNs have been shown to boost PD-1 expression [26]. In mouse spleen CD8^+^ T cells, IL-6 and IL-12 increase PD-1 expression by activating a signal transducer and activator of transcription 3 (STAT3) and STAT4, respectively [27]. Detailed mechanisms of PD-1 control, as well as the complex factors regulating the immune response, should be understood in order to boost the responsiveness of cancer patients. Novel indirect therapeutic approaches regulating PD-1 expression may expand options for cancer immunotherapy.

### 2.2. PD-L1

PD-L1, also referred to as CD279 or B7-H1, is a ligand of PD-1. PD-L1 is expressed on the surface of activated T cells, B cells, macrophages, DCs, and some non-hematopoietic cells such as epithelial cells and vascular endothelial cells [28,29]. However, in TME, tumor cells also express PD-L1 as an escape signal from the anti-tumor immune activity. PD-L1 has been shown to be highly expressed in some cancers, including renal cell carcinoma, lung cancer, ovarian cancer, and melanoma [30,31]. PD-L1 is associated with a T cell-rich immune environment, cytokines and oncogenic transcription factors, and signaling pathways.

It has been shown that inflammatory cytokine IFN-γ induces PD-L1 upregulation in ovarian cancer cells, enhancing disease progression [32]. Whereas, in acute myeloid leukemia mouse models, IFN-γ receptor 1 inhibition can reduce PD-L1 expression through the MEK/ERK and MYD88/TRAF6 pathways [33]. Moreover, IFN-γ secreted by T cells through the JAK/STAT/IRF1 axis has been shown to control the expression of PD-L1 in melanoma cells [34]. The activation of several oncogenic pathways and transcription factors are another leading cause of PD-L1 overexpression in tumor cells. Multiple oncogenic transcription factors, such as MYC, STAT3, HIF1α, HIF2α, c-JUN, NF—κB, and RELA (p65), were identified to directly regulate the transcription of PD-L1 and induce immune evasion in the TME. For example, the expression of MYC was found to be correlated with PD-L1 expression in lung cancer. In addition, high levels of HIF1α are associated with immune suppression and PD-L1 overexpression by binding on the promoter of PD-L1, suggesting that a hypoxic environment may lead to a pro-tumorigenic immune landscape as well as decreased tumor cell apoptosis [35]. Similarly, STAT3, STAT1, and RELA may also trigger PD-L1 expression via direct binding on the PD-L1 promoter [36,37,38,39].

Additionally, PD-L1 can be modulated by numerous oncogenic pathways in cancer cells. PI3K/Akt pathway is an intracellular signaling pathway that promotes metabolism, proliferation, cell survival, growth, and angiogenesis in response to extracellular signals [40]. The expression of PD-L1 can be promoted by PI3K/Akt pathway by increased extrinsic signaling or decreased negative regulators, such as PTEN [41]. It has been found that the PD-1/PD-L1 blockade in gastrointestinal stromal tumors (GIST) attenuate apoptosis of CD8^+^ T cells via regulation of PI3K/AKT/mTOR pathway [42]. Moreover, mitogen-activated protein kinase (MAPK) signaling pathway, which regulates cell proliferation, differentiation, and metastasis, is also closely associated with PD-L1 expression [43]. It has been found that MAPK pathway inhibition prevents epidermal growth factor (EGF) and IFN-γ-induced PD-L1 expression in lung adenocarcinoma cells [44]. Similarly, the treatment of anti-PD-L1 antibody decreased p-ERK and p-P38 via MAPK signaling in Hodgkin’s lymphoma cells [45]. Moreover, JAK/STAT pathway has recently been shown to be associated with PD-L1 expression in the TME of several cancers. The downregulation of JAK/STAT pathway by JAK inhibition suppressed PD-L1 expression in pancreatic and colorectal cancer cells [46,47]. In addition, many recent studies suggest that other notorious oncogenic signaling pathways, such as WNT, NF-κB, and Hedgehog pathways, may also be involved in PD-L1 expression in cancer cells [20].

## 3. CTLA-4 and Other Immune Checkpoints with Therapeutic Potentials

**Figure 2:**
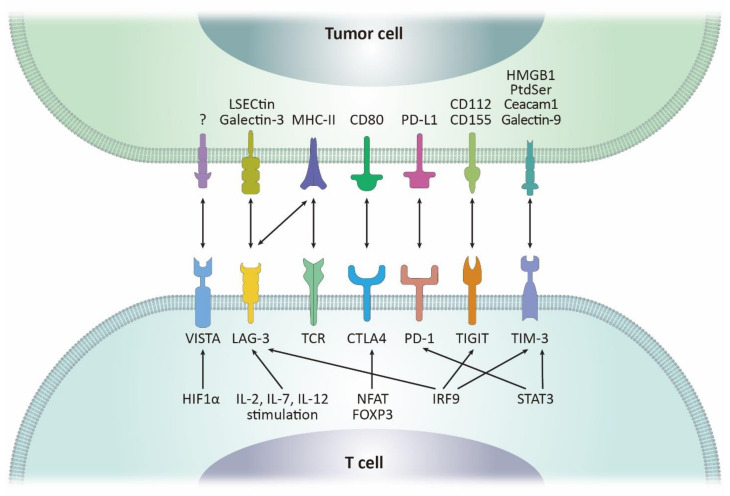
Various types of immune checkpoint receptors and ligands and their regulatory factors. Interactions of immune checkpoint receptors with ligand and their intra- and extracellular regulatory factors are indicated by arrow points. PD-1: programmed death-ligand 1; PD-L1: programmed death-receptor 1; MHC-II: major histocompatibility complex class II; CTLA-4: cytotoxic T lymphocyte antigen 4; VISTA: V-domain Ig suppressor of T cell activation; LAG-3: lymphocyte activation gene-3; TCR: T cell receptor; TIM-3: T cell immunoglobulin and mucin-domain containing-3; IRF9: IFN regulatory factor 9.

There are multiple other immune co-inhibitory molecules such as cytotoxic T lymphocyte antigen 4 (CTLA-4), lymphocyte activation gene-3 (LAG-3), T cell immunoglobulin and mucin-domain containing-3 (TIM-3), T cell immunoglobulin and ITIM domain (TIGIT), and V-domain Ig suppressor of T cell activation (VISTA). The expression of these molecules has been suggested as a potential biomarker, predicting ICI reactivity as well as an alternative target of novel ICI investigation [48,49]. Currently, there is multiple ongoing phase 1/2 clinical trials on several tumor types to determine the anti-cancer efficacy of drugs that target less studied immune checkpoints and several show positive results with high clinical efficacy and safety [50,51]. These immune checkpoints, including CTLA-4, exhibit immune responses through receptor-ligand binding, such as PD-1/PD-L1, and can be modulated through multiple intra- and extra-cellular regulatory responses (Figure 2).

### 3.1. CTLA-4

CTLA-4, also known as CD152, is expressed on the surface of activated T cells and Tregs and has been well investigated as a molecular target of the first approved ICI ipilimumab [52]. CTLA-4, a homolog of CD28, is a family of immunoglobulin-related receptors responsible for T-cell immune regulation. CD28 and CTLA-4 share a pair of ligands, CD80 and CD86, that are expressed on APCs or tumor cells [53]. However, the bindings of CD28 and CTLA-4 to their ligands mediate opposing functions in the immune system. CD28 binding to its ligands co-stimulates T cells in conjunction with T cell receptors (TCR). Conversely, CTLA-4 binds to its ligands with higher affinity than CD28 and acts as a co-inhibitory signal to interrupt early T cell activation [54,55]. The expression of CTLA-4 is tightly regulated by a number of transcription factors. Binding of NFAT to the proximal promoter of *CTLA-4* gene has been shown to upregulate CTLA-4 expression [56]. In addition, the binding of FOXP3 to the *CTLA-4* gene promoter increases histone acetylation of *CTLA-4* promoter serving as a direct activator of CTLA-4 expression [57]. In a recent study, the inhibition of NFAT/FOXP3 interaction suppressed CTLA-4 expression in T cells, demonstrating the importance of regulating NFAT and FOXP3 in anti-CTLA-4 ICI therapy [58]. The anti-CTLA-4 mAb has been widely used in the treatment of multiple solid cancers, including melanoma, renal cell carcinoma, and colorectal cancer. However, it was shown that the use of anti-CTLA-4 mAb did not deplete FOXP3^+^ cells in tumors [59]. Modulation of NFAT and FOXP3, along with inhibition of CTLA-4, may enhance the therapeutic effect of ICI therapy.

### 3.2. LAG-3

LAG-3 or CD233 is a co-inhibitory receptor expressed on activated T cells, Tregs and NK cells. The representative ligands of LAG-3 are major histocompatibility complex class II (MHC-II), and LSECtin and Galectin-3 are also known as other ligands [60,61,62]. IL-2, IL-7, and IL-12 have been reported to enhance LAG-3 expression on activated T cells, but not IL-4, IL-6, IL-10, TNF-α,β, TGF-β, or IFN-γ [63,64]. LAG3 suppresses T cell function by having a higher binding affinity for MHC-II than CD4 and it may interrupt CD4-MHC-II interaction. Furthermore, a recent study found that fibrinogen-like protein (FGL1), which is upregulated in the serum of cancer patients, can bind to LAG-3, promoting T cell inactivation [65]. A recent study showed that the triple blockade of LAG-3, PD-1, and CTLA-4 significantly enhanced antitumor immunity compared with the use of a single anti-PD-1 Ab, resulting in increased cytotoxic T cell levels and reduced Tregs and MDSCs in an ovarian cancer mouse model [66]. Several clinical trials are testing LAG-3 antagonists in combination with anti-PD-1 Ab or paclitaxel.

### 3.3. TIM-3

TIM-3 is a receptor protein also known as hepatitis A virus cellular receptor 2 (HAVCR2) and CD366 and is encoded by *HAVCR2* gene. TIM-3 is generally expressed by Th1, Th17, Tregs, and CD8+ T cells and has galectin-9, carcinoembryonic antigen cell adhesion molecule 1 (Ceacam1), high-mobility group box 1 (HMGB1), and phosphatidylserine (PtdSer) as its ligands [67]. TIM-3 been shown to be regulated by STAT3/NFIL3 and STAT1/T-bet [68]. TIM-3 enhances T cell inhibition and apoptosis and immune-suppressive activity of Tregs. Similar to PD-1, TIM-3 is associated with T cell depletion, and preclinical studies have shown that suppression of both TIM-3 and PD-1 enhances the immune-boosting effect [69,70]. It has been found that TIM-3 is highly expressed on Tregs of patients with lung cancer and hepatocellular carcinoma (HCC) patients, with 60% and 70% prevalence, respectively [71,72]. The four known ligands of TIM-3 are known to bind to different regions of TIM-3. However, the affinity or the exact binding site of TIM-3 for the ligands is yet to be elucidated. Anti-TIM-3 antibodies disrupting the binding of the ligands to TIM-3 are currently being assessed in several clinical trials and most of them are under evaluation as a potential combination partner of anti-PD-1/L1 therapy [73].

### 3.4. TIGIT

TIGIT, also identified as WUCAM, is a membrane receptor expressed on T cells and NK cells. CD155 (PVR) and CD112 (PVRL2) are the ligands of TIGIT [74]. The activation of TIGIT inhibits the function of T cells and NK cells and enhances IL-10 production of DCs, which results in T cell suppression [75]. Similar to the competitive mechanism of CTLA-4, a co-stimulatory receptor CD226 also uses CD155 as a ligand and the binding of CD226 to CD155 leads to immune activation, while TIGIT binding to CD155 leads to immune suppression [76]. However, there are not many findings on the factors that regulate TIGIT expression.

### 3.5. VISTA

VISTA, also known as B7-H5 or DD1α, is a b7 family transmembrane protein that can act both as a ligand and a receptor. VISTA is expressed in myeloid cells and T cells and is most highly expressed in Tregs and MDSCs. It has been found that p53 directly regulates the transcription of VISTA in response to DNA damage [77] and HIF-1α also induces VISTA expression [78]. An array of cancer studies has shown the expression of VISTA in lung, gastric, and ovarian cancer and its blockade decreased cancer cell proliferation [79,80,81,82].

## 4. Promising Phytochemicals Adjuvant to PD-1/PD-L1 Treatment

**Figure 3:**
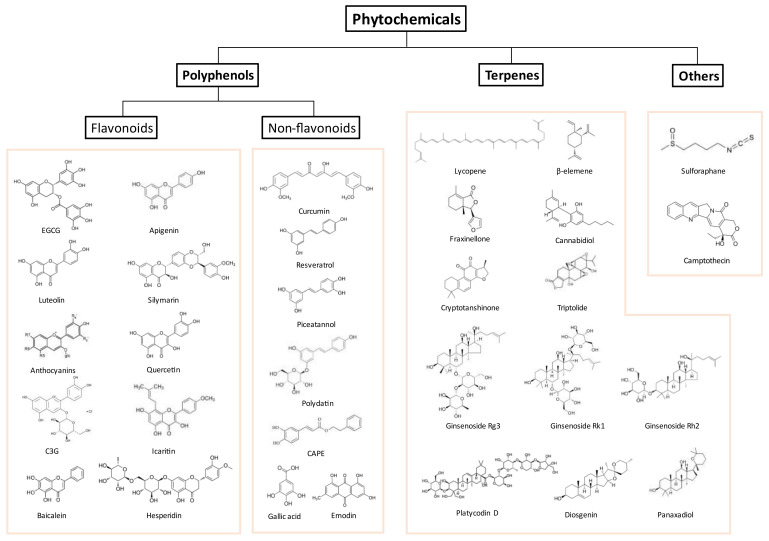
Classification of phytochemicals modulating activity of ICI according to chemical structures. Listed compounds are classified into the families of flavonoids, non-flavonoids, terpenes, and others. Flavonoids include EGCG, apigenin, luteolin, silymarin, anthocyanins, quercetin, C3G, icaritin, baicalein, and hesperidin. Non-flavonoids include curcumin, resveratrol, piceatannol, polydatin, CAPE, gallic acid, and emodin. Terpenes include lycopene, β-elemene, fraxinellone, cannabidiol, cryptotanshinone, triptolide, ginsenoside Rg3, ginsenoside Rk1, ginsenoside Rh2, platycodin D, diosgenin, and panaxadiol. Others include sulforaphane and camptothecin. EGCG: epigallocatechin gallate; C3G: cyanidin 3-*O*-glucoside; CAPE: caffeic acid phenethyl ester.

Phytochemicals are naturally occurring compounds in plants and have been reported to exert a beneficial effect on human health. Phytochemicals are mainly categorized into multiple groups such as polyphenols (e.g., resveratrol and curcumin) and terpenes (e.g., lycopene and saponins) and each group can be further divided into subgroups [83]. Various types of phytochemicals have been studied to evaluate their potential as anti-tumor therapeutics [84,85]. It has been reported that polyphenols suppress metastatic ability, proliferation, protease secretion, and angiogenesis. They may target crucial molecular mechanisms of cancer such as post-transcriptional activities, redox balance, cancer cell metabolism, and epigenetic regulations [86,87,88,89,90]. In addition to polyphenols, numerous natural compounds, including terpenes and saponins, also have immunomodulatory effects and influence the immune system in the TME by regulating immune cell activities and cytokine secretion [91,92]. In the following subsections of this review, we introduce a list of phytochemicals that may potentiate cancer immunotherapy by classification and suggest them as potential candidates for combinational use with ICI therapy (Figure 3).

### 4.1. Polyphenols

Polyphenol is a class of compounds that come in a variety of chemical configurations, from single molecules to high molecular weight polymers. Polyphenols are abundant in nature and can be found in regular human diets, including fruits and vegetables as well as flowers [93]. Polyphenols contain at least one aromatic ring, and the arrangement of aromatic rings determines whether they are flavonoids or non-flavonoids. The main structural distinction between these two groups is that non-flavonoids have one phenol ring, whereas flavonoids have two that are connected by an oxygen-containing central pyran ring [94]. Flavonoids are a group of phenolic compounds with the same backbone structure as 2-pheny1-1,4-benzopyrone (C6-C3-C6). Flavonoids are divided into flavones, flavonols, flavanones, isoflavones, flavan-3-ols, and anthocyanin. Non-flavonoids are compounds with one or multiple phenol rings, and phenolic acids, lignans, stilbenes, and curcumin are included. Both flavonoids and non-flavonoids can regulate the tumor immune microenvironment by regulating immune cells, cytokine production, and intracellular mechanisms of tumor cells (Table 1).

#### 4.1.1. Flavonoids

##### Apigenin

Apigenin is a flavone (4′,5,7-trihydroxyflavone) abundantly present in fruits, vegetables, and beverages. Apigenin is one of the widely-studied phytochemicals with various biological effects such as free-radical scavenging, anti-microbial activity, suppression of cancer cell growth, and anti-inflammatory effects [123]. Coombs et al. showed that apigenin inhibited IFN-γ-induced PD-L1 expression in human and mouse breast cancer cells by suppressing IFN-γ-induced STAT1 activation. In addition, it has been shown that luteolin, the metabolite of apigenin, also inhibited IFN-γ-induced PD-L1 expression in MDA-MB-468 human breast cancer cell line [95]. Similar results have been reported in melanoma studies. When melanoma cells were treated with apigenin or curcumin, IFN-γ-induced PD-L1 was remarkably suppressed via the inactivation of STAT1 pathway. Furthermore, apigenin or curcumin treatment on melanoma-bearing mice significantly suppressed tumor growth by inhibiting PD-L1 expression in melanoma cells showing enhanced T cell infiltration into tumor tissues. Apigenin or curcumin also inhibited PD-L1 expression in mature DCs isolated from healthy human peripheral blood mononuclear cells (PBMC). Compared to curcumin, apigenin led to greater suppressive activity on both in vitro and in vivo models [96]. Thus, altogether, inflammation-induced PD-L1 can be inhibited by apigenin.

##### Luteolin

Luteolin, a phase I metabolite of apigenin, is a flavone contained in different plants such as sage, carrots, and fennel [124,125,126]. Recently published papers have indicated the anti-cancer activities of luteolin in multiple cancer types by inducing autophagy or suppressing cancer cell proliferation [127,128]. According to a study, Coombs et al., the pre-treatment of luteolin on breast cancer cells significantly downregulated the IFN-γ-induced PD-L1 expression [87]. Although few studies on anti-cancer activities of luteolin have been published by far, it is expected that luteolin may play a role as a flavonoid in ICI therapy that regulates immune checkpoint expression.

##### Anthocyanin

Anthocyanin belongs to a class of flavonoids enriched in various plants, e.g., berries, grapes, and red onions. Anthocyanins have been demonstrated to have antioxidant, anti-inflammatory, anti-aging, anti-obesity, and anti-cancer functions [129,130,131]. Recent in vitro and in silico experiments showed that anthocyanins and their metabolites can significantly inhibit the expression of both PD-1 and PD-L1, stimulating an immune response and suppressing colon cancer progression [99]. Another study on gut microbiota has provided a new perspective on improving the efficiency of ICI therapy. The linkage between the gut microbiome and response to immunotherapy aroused an interest in microbiome profiling and its modulation. For example, it was reported that the efficacy of anti-PD-1 or anti-PD-L1 mAbs can be increased with the oral supplement of *Akkermansia muciniphila* or *Bifidobacterium* [132,133]. Wang et al. found that oral administration of bilberry anthocyanin extracts significantly enhanced the effects of the anti-PD-L1 antibody in the colon cancer mouse model. This was primarily due to the marked increase of the bacterial diversity in the anthocyanin-treated group and mice also demonstrated augmented fecal *Clostridia* and *Lactobacillus johnsonii* levels, which are known to be capable of immunoregulation [97]. Consistently, Liu et al. reported that the oral supplementation of colon cancer-bearing mice with anthocyanin or anthocyanin combo, an encapsulated anthocyanin with enhanced digestive stability, showed overrepresentation of *Lachnospiraceae* and *Ruminococcaceae.* This compositional alteration in the gut microbiome boosted the production of anti-cancer and anti-inflammatory short-chain fatty acids, especially butyrate. The anthocyanin and anthocyanin combo also increased the therapeutic efficacy of anti-PD-L1 antibody by enhancing intratumoral CD8^+^ T cell infiltration [98]. These studies have revealed the connection between intestinal flora alteration by anthocyanins and the efficacy of ICIs. Nevertheless, underlying mechanisms remain to be determined. Anthocyanins may act as potential therapeutic agents for clinical application in the future.

##### Cyanidin-3-*O*-glucoside

Cyanidin 3-*O*-glucoside (C3G) is an anthocyanin extracted from many fruits and vegetables. C3G is beneficial to the human body through antioxidant or anti-tumorigenic effects [134,135,136,137]. However, there is not much research on how C3G can affect the immune response. Mazewski et al. showed that C3G had the possibility of the non-drug treatment that inhibits PD-L1 expression. C3G significantly inhibited PD-L1 in colon cancer cell lines. C3G treatment on peripheral blood mononuclear cells (PBMCs) suppressed PD-1 expression and reduced the binding of PD-L1 and PD-1 through complex formations with C3G and PD-L1. It showed the potential of C3G to block both immune-suppressive proteins. Furthermore, it has been shown that C3G can inhibit immune checkpoints and VEGF in silico analysis [99]. These findings may imply the potential of C3G for inhibiting immune checkpoints PD-1 and PD-L1.

##### Silymarin (Silibinin)

Silymarin is a complex of flavonolignans extracted from the plant milk thistle (*Silybum marianum*) commonly used for liver disease treatment [138]. Lovelace et al. found that silymarin suppressed T cell exhaustion using PBMC samples from HIV-infected subjects. Silymarin treatment on HIV-positive CD4^+^ T cells significantly reduced the expression of CTLA-4 and PD-1, T cell exhaustion markers, showing its potential for an immune activator [100]. Silibinin is the main bioactive flavonolignan of silymarin proven to inhibit STAT3 signaling in many types of cells [139]. Several studies indicated a suppressive effect of silibinin on PD-L1 in cancer cells. Cuyàs et al. revealed that silibinin treatment on NSCLC cells significantly reduced the mRNA expression of PD-L1 and epithelial-mesenchymal transition (EMT) regulators (SNAI2, VIM, and CD44) via inhibition of STAT3 phosphorylation [101]. Silibinin also suppressed HIF-1α/lactate dehydrogenase (LDH-A)-mediated aerobic glycolysis causing inhibition of PD-L1 expression in nasopharyngeal carcinoma cells [102]. These studies offer the possibility that silymarin and its flavonolignans may also be potentially used as anti-cancer ICI therapy.

##### Epigallocatechin Gallate (EGCG)

Epigallocatechin gallate (EGCG), the most abundant catechin in green tea, is a potent antioxidant flavan-3-ols with proven anti-cancer effects in multiple cancer studies [140,141,142]. However, only a few reports on the effects of EGCG on the development of novel immunotherapeutic strategies in cancer treatment exist. Rawangkan et al. demonstrated that EGCG and green tea extract suppressed PD-L1 expression in NSCLC cells, induced by IFN-γ and EGF. Treatment with EGCG reduced IFN-γ-induced PD-L1 mRNA and protein levels through inhibition of JAK2/STAT1 signaling. Similarly, EGF-induced PD-L1 expression was reduced in EGCG-treated cells via inhibition of EGF receptor (EGFR)/Akt signaling. Using the mouse model, oral administration of green tea extract containing 14% of EGCG significantly reduced PD-L1-positive cells of lung tumors. Furthermore, the PD-L1 suppressing effect of EGCG was also evaluated in mouse melanoma cells using T cell co-culture experiment. Compared with melanoma cells only, EGCG highly reduced PD-L1 mRNA expression in T cell co-cultured melanoma cells. EGCG also recovered decreased IL-2 mRNA expression in co-cultured T cells and increased the number of T cells, indicating the restoration of T cell activity by PD-L1 inhibition [103]. These findings showed the potential of EGCG as a multi-modal PD-L1 inhibitor suppressing IFNR/JAK2/STAT1 and EGFR/Akt pathways. Further studies on EGCG with PD-1/PD-L1 inhibitors may significantly increase the ICI therapeutic effect.

##### Hesperidin

Hesperidin is a major citrus flavonoid largely found in peels of lemons and oranges. Although hesperidin is known to have anti-cancer effects in several types of cancer, little is known about hesperidin’s role in modulating immune checkpoints [143]. Recently, Kongtawelert et al. showed that in triple-negative breast cancer (TNBC) cell lines highly expressing PD-L1, hesperidin treatment suppressed cell viability and downregulated PD-L1 expression by suppressing Akt and NF-κB signaling pathways [104]. This result indicates the potential of hesperidin on anti-cancer effects that may provide insight into its role as an ICI drug.

##### Icaritin

Icaritin is a prenylflavonoid obtained from the *Epimedium genus* plant and used in traditional Chinese medicine for its tonic and stamina-boosting properties. Icaritin is used as a therapeutic for the treatment of osteoporosis and cardiovascular diseases [144,145]. Furthermore, the anti-cancer effects of icaritin, such as cancer cell growth inhibition or apoptosis, have been studied in various cancers, including hepatocellular carcinoma, glioblastoma, ovarian cancer, and cervical cancer [146,147,148,149]. With its high pharmacological properties, icaritin underwent several clinical trials with its immune modulation activities (NCT02496949) [150,151]. Two phase 3 clinical trials (NCT03236636 and NCT03236649) for the treatment of hepatocellular carcinoma are ongoing. Although icaritin is a promising anti-cancer agent, there have been few studies on immune checkpoint control. Mo et al. showed the possibility of icaritin as an immune therapeutic agent in liver cancer. Icaritin reduced cancer cell proliferation and the combined use with anti-PD-1 mAb led to more effective regulation on cancer cell death. Furthermore, icaritin suppressed PD-L1 expression by blocking IκB kinase(IKK) complex formation and decreased IKK complex formation inhibited the translocation of NF-κB p65, which acts on the promoter of PD-L1 [106]. Therefore, icaritin, which exhibits immunomodulatory activities in various carcinomas, can regulate immune checkpoint expression and has great potential for adjuvant treatment with ICI therapy.

##### Baicalein (Baicalin)

Baicalein and its conjugate baicalin are trihydroxyflavones that are found in *Scutellaria baicalensis* Georgi. Baicalein and baicalin have a role as an antioxidant, an angiogenic agent, a hormone antagonist, and an anti-inflammatory agent [152,153,154]. Many research studies suggest that baicalein and baicalin have antitumor effects, such as cancer cell apoptosis [155,156]. Although there is increasing evidence suggesting the anti-cancer properties of baicalein and baicalin, there are still few studies on ICI therapy. Ke et al. found that baicalein and baicalin significantly inhibited tumor growth and immunosuppression by regulated PD-L1 expression in liver cancer cells. The inhibition of STAT3 by baicalein and baicalin suppressed IFN-γ-induced PD-L1 expression and increased T cell-mediated liver cancer cell death. In addition, baicalein and baicalin induced the cytotoxicity of PD-1-expressing T cells by increasing IL-2 secretion [107]. This suggests the possibility of inhibiting the immune evasion of cancer cells. Thus, baicalein and baicalin that suppress HCC development are potently mediated by suppressing PD-L1 expression and enhancing host immunity.

#### 4.1.2. Non-Flavonoids

##### Curcumin

Curcumin or turmeric was firstly extracted from rhizomes of turmeric (*Curcuma longa*) in 1815. It is a bright yellow compound that belongs to a chemical class of curcuminoid. Curcumin has been used for medical treatment, seasoning, and fabric dyeing in Asian countries for more than 2000 years [157]. Curcumin is proven to have bioactivity and pharmaceutical properties. Curcumin can exert anti-cancer, anti-inflammatory, antioxidant, and anti-microbial functions, etc. [158]. Recent research has demonstrated that curcumin exhibits positive effects on a wide range of diseases, among which cancer is the most extensively studied. Curcumin is capable of targeting multiple pathways to affect cancer development and progression, becoming a potential anti-cancer agent in clinical use. Curcumin, solely or in combination with other treatments, has been validated to suppress the tumor growth or metastasis in colorectal cancer [159], prostate cancer [160], pancreatic cancer [161], breast cancer [162] and many other cancer types. Recent studies have reported that curcumin may be a potential agent for improving the response of immune therapy.

Activation of NF-κB and STAT3 signaling pathways in cancer cells and immune cells is crucial in inducing immunosuppression. Previous evidence has shown that curcumin can inhibit NF-κB and STAT3 signals to attenuate tumor progression. In tongue squamous cell carcinoma, curcumin was shown to partially reverse immune suppression via inhibiting STAT3 pathway-mediated PD-L1 expression and decreasing the recruitment of regulatory T cells (Tregs) and myeloid-derived suppressor cells (MDSCs) [108]. COP9 signalosome complex 5 (CSN5) is a deubiquitinase modulating the ubiquitination of PD-L1. It was reported that TNF-α can promote the transcription of CSN5 through activation of the NF-κB signaling pathway. CSN5 then binds to and deubiquitinates PD-L1, thus enhancing expression of PD-L1. Lim et al. reported that curcumin was able to augment the stabilization of PD-L1 and enhance the anti-cancer immunity through abating the activity of CSN5. These results herald that curcumin may potentially act as an adjuvant to enhance the efficacy of immune therapy [109]. Moreover, Hayakawa et al. found that curcumin administration in the colon cancer mouse model significantly increased the tumor antigen-specific CD8^+^ T cell induction as well as enhanced the T cell stimulatory activity of dendritic cells (DCs) by downregulating NF-κB and STAT3 signaling pathways. They also revealed that combining curcumin with an anti-PD-L1 antibody had a synergistic tumor-suppressive effect [110]. Shao et al. established mouse models bearing subcutaneous or metastatic bladder cancer and found that bisdemethoxycurcumin, an analog of curcumin, in combination treatment with an anti-PD-L1 antibody could significantly upregulate the secretion of IFN-γ and granzyme B and decrease the number of MDSCs, which successfully boosted immune response and prolonged mouse survival [113].

Anti-cancer therapy with curcumin is evolving beyond the co-treatment with immunotherapy drugs. Drug delivery systems, such as a formulation or device, are engineered to improve the targeted delivery of the drugs. Extracellular vesicles and nanoparticles have emerged as efficient drug delivery systems for cancer treatment [163]. Recently, Xiao et al. developed a dual pH-sensitive core-shell structural nanodrug with anti-PD-1 mAb on the surface and curcumin in the core [111]. Solid tumors usually have an enhanced permeation and retention (EPR) effect on nanodrugs [164]. However, this passive accumulative pattern is not as effective as expected due to the heterogeneity of solid tumors [165]. The anti-PD-1 mAb-conjugated nanocarrier overcomes this issue by selectively binding to circulating PD-1^+^ T cells following their infiltration into the solid tumor. The dual pH sensitivity enabled the subsequent drug secretion inside the tumor. The anti-PD-1 mAb would be firstly released in response to the weak acidic TME (pH ~6.5), which also allowed easy internalization by converting the surface charge from negative to positive. The anti-PD-1 mAb released in the tumor extracellular matrix could induce the tumor-suppressive effects of cytotoxic T cells. Curcumin inside then would be rapidly released in the lysosomal microenvironment (pH ~5.5), which could reduce the recruitment of Treg and promote the infiltration of anti-tumor T cells through inhibition of NF-κB pathway and downregulation of cytokine secretion [111]. In contrast, curcumin-induced expression of CTLA-4 was reported in mouse spleen-derived CD4^+^CD25^+^ Tregs [112]. This indicates that curcumin has pleiotropic activities mediating both inhibitory and stimulatory effects on Tregs by differently regulating immune checkpoints. Although a growing body of evidence has revealed the possible immunomodulatory roles of curcumin, there is still a lack of comprehensive mechanistic studies and clinical trials. More in-depth research is warranted before clinical translation.

##### Resveratrol

Resveratrol (RSV), a type of natural phenol stilbene, was first discovered and identified in the roots of white hellebore by Michio Takaoka in 1939 [166]. RSV is naturally synthesized in many plants in response to the fungal infection or injury by environmental factors such as ultraviolet light [167]. RSV, one of the most well-known phytochemicals, has long been used in China and Japan as a traditional medicine [168,169]. It has been reported to have various biological effects such as antioxidant, free-radical scavenging, cardioprotective, neuroprotective, anti-microbial, and anti-cancer activity [170]. In addition, RSV also has a regulatory effect on the immune system. Numerous studies have shown that RSV can directly and indirectly control cancer cells and immune cells, enabling the regulation of immune responses in various types of cancer.

Since PD-L1 is a type I transmembrane glycoprotein, glycosylation status plays a crucial role in determining the stability and function of PD-L1 protein [171]. It has been previously reported that RSV disrupts *N*-linked glycosylation of proteins in ovarian cancer cells [172]. In a recent study, RSV facilitated the accumulation of an abnormally glycosylated form of PD-L1 by disrupting *N*-linked glycosylation in breast cancer cells and inhibited cell membrane localization. Furthermore, computer simulations predicted the capacity of RSV to induce PD-L1 dimerization that may inhibit PD-1/PD-L1 interaction directly. Modulation of PD-L1 glycosylation and dimerization enhanced T cell cytolytic activity against cancer cells [114]. Also, RSV antagonized thyroxine-induced PD-L1 expression. Thyroxine, a thyroid hormone, resulted in the downregulation of pro-apoptotic factor BAD and upregulation of PD-L1 and proliferative factor CCDN1 in oral cancer cell lines. RSV treatment reversed the effects of thyroxine in oral cancer cells and reduced PD-L1 expression and nuclear accumulation [115]. Moreover, Zhang et al. confirmed the synergetic anti-cancer effects of RSV with anti-PD-1 antibody in mouse in vivo models of ovarian cancer. RSV treatment on mouse model transperitoneal injected with ovarian cancer cells significantly induced DCs and CD8^+^ T cells in tumor tissues, suppressing tumor progression. While anti-CD8 antibody co-treatment restored the tumor growth, the co-treatment of RSV with anti-PD-1 significantly suppressed the tumor growth, stimulating both DCs and cytotoxic T cells [116]. Conversely, other research has shown that both RSV and piceatannol treatment on breast and colorectal cancer cells expressing low levels of PD-L1 increased surface expression of PD-L1 via NF-κB signaling pathway and histone modification. RSV and piceatannol-induced PD-L1 also promoted cancer cell apoptosis via DNA damages [117]. Moreover, RSV can also modify CTLA-4 expression in Tregs. Weng et al. found that RSV treatment significantly increased CTLA-4 expression in mouse spleen and thymus-derived CD4^+^CD25^+^ Tregs [118]. Similarly, CTLA-4 expression and a number of peripheral blood Tregs were upregulated in high-fat diet mice by RSV supplementation [119]. However, there was no direct evidence of RSV modulating CTLA-4 expression in cancer studies.

Many studies have suggested that RSV may modulate immune checkpoint expression in immune cells of TME and nontumor models. RSV-induced CTLA-4 expression in Tregs and regulation of PD-L1 expression in cancer cells suggest the need for further research on RSV and cancer immunotherapy.

##### Piceatannol

Piceatannol is a naturally occurring biotransformed product of resveratrol. Piceatannol has not been studied much compared to resveratrol but has exhibited more diverse biological activities than resveratrol [173]. Lucas et al. found that piceatannol increased PD-L1 expression and combined use with resveratrol synergistically upregulated PD-L1 in low PD-L1 breast cancer and colon cancer cells. The combined treatment-induced PD-L1 expression was suppressed by using histone modification inhibitor and NF-κB pathway inhibitor, respectively. Tumor cell survival was also decreased with the combined treatment through DNA damage and cell cycle arrest [117]. Regarding the evidence that modifying histone acetylation and deacetylation can regulate immune response and PD-L1 expression [174,175], combined treatment of resveratrol and piceatannol may elicit PD-L1 upregulation through NF-κB signaling mediated by histone modification. This result provides the possibility that anti-PD-L1 therapy may be used in the group of cancer patients with low PD-L1 expression.

##### Polydatin

Polydatin, or piceid, is a natural precursor of resveratrol. It is a prominent bioactive compound extracted from the Chinese herb *Polygonum cuspidatum* [176,177,178]. Polydatin is shown to have antitumor effects such as apoptosis or anti-proliferation in various cancer types [179,180,181]. However, the mechanism of immune checkpoint regulation by polydatin in TME is unknown. In a recent study, polydatin promoted apoptosis and inhibited the proliferation of colorectal cancer cells by regulating the miR-382/PD-L1 axis. Since miR-382 binds to the transcript of PD-L1, the overexpression of miR-382 inhibited the expression of PD-L1. Polydatin treatment on colorectal cancer cells upregulated miR-382 and suppressed PD-L1 expression [120]. Thus, polydatin is expected to be a potential compound as a novel ICI therapy drug.

##### Caffeic Acid Phenethyl Ester (CAPE)

Caffeic acid phenethyl ester (CAPE), a hydroxycinnamic acid, is an important active component of honeybee propolis extract and has been used in traditional medicine for years. To date, many studies have reported that CAPE has many biological properties such as anti-inflammatory, antioxidant, and anti-cancer effects [182,183]. Fang et al. found that Epstein-Barr virus (EBV) infection in NPC cells induced PD-L1 expression through latent membrane protein 1 (LMP1) and IFN-γ pathway, respectively. The treatment of CAPE, as an inhibitor of NF-κB, remarkably decreased the expression of PD-L1 in EBV-positive nasopharyngeal carcinoma (NPC) cell line and LMP1-overexpressed normal nasopharyngeal epithelial cell line [121]. These results indicated a therapeutic potential of CAPE for supportive use with ICI treatment by suppressing LMP1-induced PD-L1 expression through inhibiting NF-κB pathway.

##### Gallic Acid

Gallic acid (GA) is a phenolic acid extracted from natural plants, fruits, and green tea and exhibits antioxidant, anti-inflammatory, and anti-cancer activities [184,185,186,187,188]. Recently, GA also has been found to have immunomodulatory effects in cancer cells. In NSCLC cells, GA functioned as an antagonist and suppressed EGF binding on EGFR, resulting in PI3K/AKT pathway inhibition. GA-dependent inhibition of PI3K/AKT upregulated the expression of p53 and miR-34a, which is induced by p53 and inhibits PD-L1 expression. In addition, GA and anti-PD-1 mAb co-treatment decreased the expression of PD-L1 and activated the T-cell-mediated immune responses such as IFN-γ increase [122]. Thus, GA has the potential of immune checkpoint regulation and for use as an ICI therapy agent.

##### Emodin

Emodin is a well-known anthraquinone that can be isolated from several Chinese herbs, including Rhubarb (*Rheum palmatum*). Emodin has been shown to have anti-inflammatory and anti-cancer properties such as cancer cell apoptosis, proliferation inhibition, and chemotherapy sensitization [189,190,191,192]. However, there are relatively few reports of emodin on immune checkpoint modulation. Using breast cancer cells, Lim et al. observed that the treatment of emodin as an anti-inflammatory supplement with NF-κB inhibitory role attenuated TNF-α-mediated PD-L1 stabilization which resulted in the upregulation of plasma membrane PD-L1 expression. The decrease in PD-L1 expression led to a decrease in PD-1 binding, enhancing T cell-mediated tumor cell death [109]. Given these results, emodin can regulate the PD-L1 plasma membrane expression via NF-κB signaling pathway and further validation of its activities on other cancer cells may be needed.

### 4.2. Terpene

Terpenes is a wide group of natural compounds and is also known as terpenoid or isoprenoid. Terpenes are classified as monoterpenes, diterpenes, triterpenes, tetraterpenes, and sesquiterpenes base on the organization and number of isoprene units [193]. Lycopene, cannabinoids, and saponins are the well-known terpenes. Terpenes have been shown to exert multiple beneficial effects on human health including antiviral, antidiabetic, antidepressant, and anti-cancer activity [194,195,196]. Terpenes also enhance the immune system by regulating cytokine secretion or T cell reactivity in several infectious diseases and cancers [197]. Recently, it has been confirmed that several terpenes can regulate ICI inhibitor reactivity and PD-1/PD-L1 expression through molecular mechanisms (Table 2).

#### 4.2.1. Lycopene

Lycopene is a naturally occurring red carotenoid (tetraterpenoid) found in red to pink fruits and vegetables such as tomatoes. Lycopene has been extensively studied for many years with its potential health benefits, especially in cancer and cardiovascular disease [217,218,219]. A recent study reported that lycopene can synergistically function with anti-PD-1 therapy and modulate PD-L1 expression in the mouse lung cancer model. The combined treatment of lycopene and anti-PD-1 antibody on a lung cancer cell injected mouse reduced tumor volume and weight by enhancing tumor cell apoptosis. Additionally, lycopene reduced IFN-γ-induced PD-L1 expression in lung cancer cells by activating JAK2/STAT3 signaling and inhibiting AKT. In contrast, there was no change in PD-L1 expression by lycopene treatment without IFN-γ [92]. This study suggests that lycopene may be beneficial in cancer patients with PD-L1 expression and high IFN-γ levels. However, the mechanism of this study differs from other studies in that IFN-γ-induced PD-L1 was inhibited through JAK2 activation, not JAK2 inhibition, indicating that further research is warranted.

#### 4.2.2. Fraxinellone

Fraxinellone is a terpenoid found in the root of *D. dasycarpus* and has been widely used as a drug for pesticidal and cancer treatment. Xing et al. found that fraxinellone dose-dependently inhibited PD-L1 expression in multiple cancer cell lines, including lung, cervical, colon, and hepatic cancers. Especially in lung cancer cell lines, fraxinellone significantly reduced STAT3 activation and HIF-1α protein synthesis via JAK/Src, mTOR/p70S6K/eIF4E and MAPK pathways. As STAT3 and HIF-1α are the transcription factors that induce PD-L1 transcription, fraxinellone treatment effectively decreased PD-L1 levels and reduced the tumor growth in an in vivo xenograft mouse model. Furthermore, fraxinellone-induced PD-L1 reduction decreased angiogenesis in endothelial cells via VEGF and MMP-9 [198]. Fraxinellone suppressed PD-L1 expression in multiple cancer cells through modulating several oncologic pathways. However, this does not provide a specific target for fraxinellone. More research into fraxinellone’s mechanism of action is needed to promote its clinical application.

#### 4.2.3. β-elemene

β-elemene is a sesquiterpene and the main active compound among monomer forms of elemenes. β-elemene is being clinically tested in various diseases, including cancers as an adjuvant treatment with accumulated preclinical studies [220,221]. Nevertheless, studies on β-elemene modulating immune checkpoint and related molecular mechanisms are rare. Liang et al. found that β-elemene suppressed PD-L1 expression and cancer cell proliferation in esophageal cancer cells. In both in vitro and in vivo experiments, β-elemene consistently inhibited Akt activation as well as expression of its downstream molecule, PD-L1 [199]. Although the general mechanism by which β-elemene suppresses tumors remains unclear, several studies have drawn attention to Akt as a target [222,223,224,225]. Akt has been studied to promote PD-L1 expression, and β-elemene treatment for high PD-L1 expressing cancers may help awaken anti-tumor immunity.

#### 4.2.4. Cryptotanshinone

Cryptotanshinone (CT), also called tanshinone C, is a quinoid diterpene. CT is a major tanshinone extracted from the roots of *Salvia miltiorrhiza* (Danshen) [226]. CT has been found to have clinical effects in chronic diseases, such as blood and vessel-related disorders, Alzheimer’s disease, and cancers by its pharmacological activities [227]. Recently, it has been reported that CT also has an immunomodulatory role in ICI therapy. Through a mouse in vivo study, Han et al. found that CT treatment on an HCC bearing mouse significantly slowed down the tumor growth. Combined treatment with CT and anti-PD-L1 antibody completely inhibited the tumor growth. The re-inoculation of the same cancer cells did not develop tumors, indicating the development of long-term immunity. Furthermore, the combined treatment on HCC bearing mice increased the tumor-infiltrating CD8^+^ T cells and promoted lymph nodes retaining memory and effector CD8^+^ T cells, suggesting additional anti-tumor efficacy of CT on anti-PD-L1 [200]. Overall, CT is recognized as an outstanding synergetic molecule on ICI therapy with its induction of long-term tumor-specific immunity.

#### 4.2.5. Triptolide

Triptolide, a diterpene triepoxide from the traditional Chinese herb *Tripterygium wilfordii*, has been reported to exert anti-inflammatory and immunosuppressive effects in different diseases suppressing T cell activation and IFN-γ secretion [228,229]. In addition, preclinical studies indicated that triptolide exhibits anti-tumor effects in a number of cancers, including oral, pancreatic, and breast cancer [230,231,232]. Based on previously published papers, there is increasing evidence that triptolide can effectively regulate IFN-γ-induced PD-L1 expression in various cancer cells. Liang and Fu found that triptolide suppressed IFN-γ-induced PD-L1 expression in the surface of breast cancer cell line. [201]. Furthermore, triptolide inhibited IFN-γ induced PD-L1 expression in glioma cell lines and reversed glioma cell-induced CD4^+^ T cell inhibition [202]. Triptolide reduced IFN-γ secretion and inhibited the IFN-γ-related JAK2-STAT1 pathway in oral cancer cells, decreasing PD-L1 expression and cancer cell proliferation [203]. Multiple preclinical evidence has shown a correlation between IFN-γ and PD-L1 in various carcinomas, which indicates the clinical research potential of triptolide.

#### 4.2.6. Cannabinoid

Cannabinoid is a group of chemical compounds found in the plant cannabis. Cannabidiol (CBD) and tetrahydrocannabinol (THC) are the best-known phytocannabinoids. CBD is a non-intoxicating compound with therapeutic benefits and THC makes an individual intoxicated. Numerous studies extensively showed that cannabinoids have antitumor and symptom management benefits in cancer patients [233,234]. Recent findings suggested an immunomodulatory role of cannabinoids. CBD and THC reduced PD-L1 expression in pancreatic cancer cells and pancreatic stellate cells through inhibition of p-21 activated kinase 1 (PAK1), which is an important effector protein of Kras [204]. However, in a retrospective observational study on patients with advanced melanoma, NSCLC, and renal clear cell carcinoma, the use of cannabis products during immunotherapy (nivolumab) reduced the response rate (RR) and did not significantly affect the progression-free survival (PFS) or overall survival (OS) [235]. Since it is a retrospective study according to the use of cannabis products, further studies are needed to confirm a direct correlation between cannabidiols and cancer progression at various stages.

#### 4.2.7. Saponins

Saponins comprise a large family of structurally related compounds containing a steroid or triterpenoid aglycone (sapogenin) linked to one or more oligosaccharide moieties. They are characterized by their hemolytic activity and foaming properties and are responsible for imparting a bitter taste and astringency to plant materials containing a high concentration of saponins. Saponins have been reported in Panax species (ginseng) and many edible legumes [236]. Several studies have demonstrated the beneficial effects of saponin on human health, including anti-cancer, anti-obesity, and anti-oxidative effects, including hypocholesterolemic effects [237].

Ginsenosides are triterpenoid saponin found exclusively in ginseng. In a recent study using enzyme-linked immunosorbent assay (ELISA), 8 out of 12 ginsenosides showed inhibitory effects on PD-1/PD-L1 interactions at 35% at the maximum concentration. Among them, Ginsenoside Rg3 and Compound K exhibited the highest inhibitory effects [238]. Furthermore, ginsenoside Rg3 induced apoptosis in cisplatin-resistant human lung cancer cell line (A549) via inhibition of PD-L1, AKT, and NF-kB p65 [205]. Similarly, in lung cancer cell lines (A549 and H1299), ginsenoside Rh2 repressed EGFR, PI3K/AKT, autophagy, and cisplatin-induced PD-L1 expression via inhibiting superoxide generation [206]. Similarly, Ginsenoside Rk1 induced apoptosis and suppressed PD-L1 expression by inhibiting NF-κB and Bcl-2 in lung cancer cells (A549) [207]. Platycodin D is a triterpenoid saponin isolated from *Platycodon grandifloras*. Interestingly, Huang et al. found that Platycodin D reduced PD-L1 protein levels in lung cancer cells via triggering PD-L1 secretion into the cell culture medium, which is independent from saponin’s hemolytic mechanism [209]. In colon cancer cells, panaxadiol, another subtype of ginsenoside, inhibited PD-L1 expression and tumor cell proliferation by suppressing HIF-1α and STAT3 [208]. Triterpenoid saponins isolated from *Anemone flaccida* Fr. Schmidt induced the apoptosis of HCC cells by blocking the activation of PD1/PD-L1, ERK1/2, p38 MAPK, JNK, and STAT3 signaling pathways and altering the metabolism of cancer [210]. Moreover, diosgenin, a steroidal saponin, modulated intestinal microbiota and facilitated antitumor immunity through increased CD4^+^/CD8^+^ T-cell infiltration and IFN-γ in melanoma-bearing C57BL/6 mice. Combined administration of diosgenin with PD-1 antibody enhanced tumor cell apoptosis by T cell immune response [211].

### 4.3. Other Natural Compounds and Plant Extracts

#### 4.3.1. Sulforaphane

Sulforaphane is a natural sulfur-containing isothiocyanate derived from certain species of the *Brassica* vegetable family including broccoli. Therapeutic effects of sulforaphane on various diseases such as chronic inflammatory diseases, skin diseases, and cancers are well-demonstrated by multiple studies, including clinical studies [239]. Kumar et al. found that healthy donor-derived CD14^+^ monocytes cultured in glioblastoma-conditioned media (GCM) induced monocytic MDSC transformation along with overexpression of PD-L1. Sulforaphane treatment on GCM-treated monocytes promoted T cell proliferation by increasing the transformation of monocytes into mature DCs instead of MDSC. Sulforaphane also suppressed PD-L1 expression in GCM-treated monocytes in a dose-dependent fashion [91]. Considering the low permeability of ICIs to blood brain barrier, this finding suggests that the combined application of sulforaphane to glioblastoma ICI therapy may provide a significant therapeutic effect.

#### 4.3.2. Camptothecin

Camptothecin is a pentacyclic alkaloid isolated from the bark of *Camptotheca acuminata*. Camptothecin exerts antitumoral activity as a topoisomerase 1 inhibitor and its derivatives, topotecan and belotecan, are clinically available anti-cancer drugs. Camptothecin has been studied to be well suited for cancer immunotherapy with its cytotoxic effect and immune cell modulatory ability [240]. Tai et al. found that camptothecin dose independently induced PD-L1 expression in colon cancer cell lines. Moreover, camptothecin upregulated the secretion of cytokines that modulate the attraction, migration, and functions of immune cells [212]. In addition, DNA double-strand break (DSB) induced by camptothecin or other DSB-inducing agents enhanced PD-L1 expression in osteosarcoma, lung cancer, and prostate cancer cell lines. DSB-dependent PD-L1 upregulation was mediated via ataxia telangiectasia mutated (ATM)/ataxia telangiectasia and Rad3-related protein (ATR)/Chk1 activation and STAT1/3-IRF1 pathway. BRCA2 depletion also enhanced DSB-induced PD-L1 upregulation [213]. These studies revealed the underlying mechanisms of PD-L1 expression and showed that DNA instability caused by camptothecin or DNA targeting agents (ex, platinum) regulates the expression of PD-L1 through DNA repair-related pathways.

#### 4.3.3. Plant Extracts

*Rhus verniciflua* Stokes (RVS), commonly known as Chinese lacquer tree, has been used in traditional Korean herbal therapy containing numerous bioactive phytochemical constituents [241]. The anti-cancer and antiangiogenetic effects of RVS extracts have been demonstrated in Lewis lung carcinoma cell with in vitro mouse model and allergen-removed RVS extracts have successfully treated two patients with advanced renal carcinoma in a clinical observational study [242,243]. Recently, Li et al. found that the compounds isolated from RVS extract exhibit inhibitory effects on the binding of PD-1/PD-L1 and CTLA-4/CD80 by using competition ELISA. The ELISA analysis of RVS bioactive constituents on immune checkpoint interaction showed that, among 20 major identified compounds from RVS extract, eriodictyol, fisetin, liquiritigenin, and quercetin have inhibitory effects on PD-1/PD-L1 molecular binding. Notably, eriodictyol and fisetin exhibited substantial blocking effects. From another study, quercetin, a flavonoid found in many fruits and vegetables, have also been demonstrated to suppress IFN-γ-dependent PD-L1 expression in melanoma cells [105]. For CTLA-4/CD80 binding, protocatechuic acid exhibited the most potent blocking efficiency among 20 RVS extract-derived compounds, followed by caffeic acid, taxifolin, and butin [214]. Although the results were only analyzed with ELISA, they show the potential of conducting in vivo experiments, proposing several probabilities on ICI therapy with phytochemicals. In addition, *Anoectochilus formosanus*, a species of Jewel Orchid which is used to treat bruises and poisonous snake bites, also showed immunomodulatory effect in cancer. Ho et al. found that *A. formosanus* extract has free radical scavenging capacity and the treatment of AF on oral cancer cells inhibited PD-L1 expression and its protein accumulation [215]. Similarly, the treatment of *Prunus mume* extract MK615 improved survival rate and decreased PD-L1 and NF-κB expression in a melanoma-bearing mouse model [216].

## 5. Discussion

Phytotherapy is defined as a medical science that uses plant extracts or phytochemicals to treat diseases or improve health. Antioxidant, anti-inflammation, and angiogenesis activity of natural compounds are also effective in the treatment of cancer. With shown biological activities in the human body, phytochemicals can function as a poison or a medicine, depending on its type and usage. Representatively, the Pacific yew tree was known as a toxic plant, but paclitaxel isolated from the bark of the Pacific yew tree is widely used as an essential anti-cancer agent in many carcinomas today. Modern pharmacology reveals that phytochemicals isolated from medicinal plants are used as a compound itself or function as lead compounds by using the intrinsic principle of action of natural compounds.

Phytochemicals are naturally found in many plants, and their consumption is generally believed to give health benefits. Humans have adapted to toxic compounds due to long-term plant intake, and long-term administration of low concentrations of phytochemicals positively affects health and longevity through the hormesis effect [244]. The compounds listed in this review have been well-studied for their anti-cancer effects for decades. Resveratrol, curcumins, sulforaphane, EGCG, and lycopene are representative phytochemicals that are actively undergoing clinical trials for cancer [245]. However, indiscriminate use of phytotherapy is dangerous and not all phytochemicals are safe to consume. Intake of certain phytochemicals, such as capsaicin, phytoestrogens, and amygdalin, may act as carcinogens or tumor promoters [246].

The majority of phytochemicals introduced in this review regulate the expression of PD-1/PD-L1 in several carcinomas through the expression of intracellular molecules, posttranslational modifications, and regulation of signaling pathways and have a synergetic effect on ICI therapy in vitro and in vivo. This mechanism may also affect the expression of other less studied immune checkpoints such as LAG-3 and TIM-3. For example, it has been studied that apigenin regulates the expression of PD-L1 through STAT1 signaling and that TIM-3 is also regulated by STAT1. This suggests that the adjuvant use of phytochemicals can be effective when used in combination with ICI inhibitors with high side effects.

Phytochemicals in herbs and fruits produce health benefits, but questions regarding their bioavailability remain to be answered. For example, resveratrol, a representative polyphenol, showed significant in vitro therapeutic potentials but the bioactivity was low when used orally [247]. Whereas non-polyphenolic compounds such as lycopene and SFN have been shown to be highly bioactive [248,249]. Bioavailability is a sum of bioaccessibility and bioactivity. The bioavailability of phytochemicals is affected by several factors including metabolism, transport, and assimilation [250]. Numerous recent studies have shed light on the link between microbiota and phytochemicals. The gut microbiota can regulate host metabolisms through modulating fatty acid oxidation, short-chain fatty acid synthesis, and gut hormone release [251,252]. Orally absorbed phytochemicals pass through the digestive tract and interact with microbiota. Then, phytochemicals are degraded by microbial enzymes that induce bio-transformation such as hydrolysis, decarboxylation, and deamination. The bioavailability of phytochemicals varies by their subtypes. Relative to non-polyphenols, the majority of dietary polyphenols do not undergo intestinal absorption and are absent in urine [253]. Around 90–95% of ingested polyphenols accumulate in the large intestine where the microbiota facilitate degradation and absorption of ingested compounds [254]. For example, the polyphenolic compound baicalin can only be absorbed after hydrolyzed into baicalein by microbiota [255]. The comprehensive influence of the gut microbiome on phytochemicals has been reviewed by Dey [256]. The human gut microbiota is crucial for regulating phytochemical bioactivity. Therefore, understanding how microbial factors affect phytochemicals may be critical for translation into clinical settings.

Several preclinical and clinical trial data suggest that phytochemicals provide therapeutic benefits to the human body. For instance, resveratrol has been studied in clinical trials for a variety of diseases, including cancer, neurological disorders, cardiovascular diseases, and diabetes [257]. In particular, resveratrol has been shown to be effective in metabolic diseases, including obesity and diabetes, by regulating mitochondrial health and turnover [169,258,259]. Furthermore, clinical trials on inflammation, cardiovascular diseases, metabolic disorders, and cancers have revealed the pharmacological properties of curcumin with only minor side effects [260]. Icaritin has been used in several clinical trials due to its excellent stability and therapeutic effect. Clinical trials (NCT02496949) have been conducted on its immunomodulatory activity in solid carcinomas, including HCC [150,151]. Several clinical trials using phytochemicals have been conducted for various diseases or immune-related activity, but there are currently no clinical trials for phytochemicals with ICI monotherapy or combination agents with other immunomodulatory anti-cancer agents. This may be due to the poor pharmacokinetics and pharmacodynamics features of a large number of phytochemicals [261]. Current ICI therapy is used alone or combined with conventional chemotherapy in patients with certain cancer types, while severe side effects can be accompanied during combination therapy. Phytochemicals can be a good option as an adjuvant for ICI therapy for fewer side effects. Improving the bioavailability of phytochemicals through delivery systems such as nanodrugs may potentially enable the clinical translation of phytochemicals in immunotherapy.

Due to the nature of immunotherapy that can cause high side effects, a strategy both maximizing the therapeutic effect and attenuating the adverse effects is needed. The combined therapy of ICI with a low-toxic molecule, which has been well studied with the molecular mechanism in physiological and pathological processes in humans, can give synergetic effects through precise molecular targeting.

However, in the case of resveratrol and camptothecin, it has been shown that the expression of immune checkpoints is regulated differentially depending on the carcinoma. This indicates that natural compounds may act in a disease-specific way and can have detrimental effects in some cases of cancer patients. Therefore, better understanding and characterization of each phytochemical through in-depth preclinical studies must be required before proceeding to the clinical stages of testing. Instead of chemotherapeutic reagents of high cytotoxicity, the combined use of phytochemicals that enable precise targeting of the immune system and tumor molecular activity may be a safer option to overcome the limitations of current ICI therapy.

## 6. Conclusions

**Figure 4:**
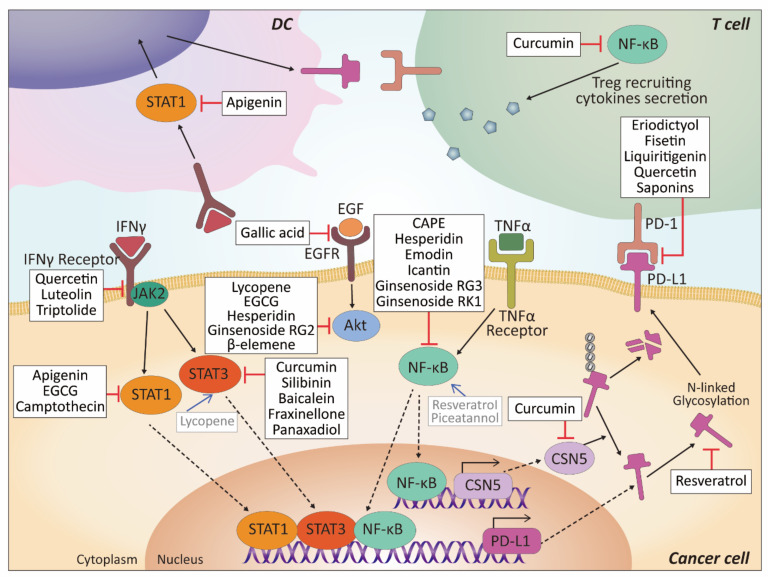
Schematic representation of signaling pathways regulating PD-L1 expression in cancer cell, dendritic cell (DC), and T cell targeted by bioactive phytochemicals. Phytochemicals modulate therapeutic effects of ICI and expression of PD-L1 through the regulation of multiple signaling pathways, including IFN-γ/JAK/STAT, EGFR/Akt, and TNF-α/NF-κB signaling pathways. Many phytochemicals regulate PD-L1 expression by targeting pathways related to PD-L1 transcription and certain phytochemicals inhibit glycosylation of PD-L1 protein or PD-1/PD-L1 binding. Phytochemicals are listed in white or grey boxes. Arrows indicate activations; blunt-ended lines indicate inhibitory effects; dotted arrows indicate translocations.

This paper reviews the connections between ICI therapy and common dietary phytochemicals that have been widely used in medicine exhibiting antioxidant, anti-inflammatory, and anti-cancer effects through the regulation of intracellular biological systems. A number of phytochemicals regulates the expression of PD-L1 through the regulation of transcription factors, such as STAT and NF-kb, protein ubiquitination, and glycosylation (Figure 4). In addition, phytochemicals may inhibit the interaction of PD-1/PD-L1 through direct binding or modulating the gut microbiota. Various phytochemicals exert the immunogenic death of tumor cells. Thus, they may have a solid potential to be a natural combination partner of anti-PD-1/PD-L1 therapy. However, further mechanistic and clinical studies are warranted to characterize the most effective phytochemicals with anti-tumor immunity.

## Figures and Tables

**Table 1:** List of polyphenolic compounds (flavonoids and non-flavonoids) and their effects with mechanisms on immune checkpoints modulation.

Compound	Effect on ICI	Mechanism of Effect	Cell Type/Model	Refs
Apigenin	↓PD-L1 expression	Inhibits PD-L1 expression and enhances T cell proliferation via downregulation of IFN-γ-induced STAT1 activation	Breast cancer cells	[95]
	↓PD-L1 expression	Inhibits PD-L1 expression in melanoma cells via downregulation of IFN-γ-induced STAT1 pathway activation Inhibits PD-L1 expression in human mature DCs	Melanoma cells and mouse in vivo model Human PBMCs	[96]
Luteolin	↓PD-L1 expression	Inhibits the IFN-γ dependent PD-L1 upregulation	Breast cancer cells	[87]
Anthocyanins	Synergistic effect with anti-PD-L1 Ab	Modulates gut microbiota especially *Clostridia* and *Lactobacillus johnsonii*	Mouse colon cancer in vivo model	[97]
	Synergistic effect with anti-PD-L1 Ab	Modulates gut microbiota especially *Lachnospiraceae* and *Ruminococcaceae*	Mouse colon cancer in vivo model	[98]
Cyanidin-3-*O*-glucoside	↓PD-1 and PD-L1 expression	Inhibits PD-1 expression in PBMCs, and PD-L1 and VEGF expression in colorectal cancer cells	Human PBMCs and colorectal cancer cells	[99]
Silymarin (Silibinin)	↓CTLA-4 and PD-1 expression	Inhibits T cell functions	HIV-positive human CD4+ T cells	[100]
	↓PD-L1 expression	Suppresses the mRNA expression of PD-L1 and EMT regulators via inhibition of STAT3 phosphorylation	NSCLC cells	[101]
	↓PD-L1 expression	Suppresses HIF-1α/LDH-A-mediated aerobic glycolysis	Nasopharyngeal carcinoma (NPC) cells	[102]
Epigallocatechin gallate (EGCG)	↓PD-L1 expression	Reduces IFN-γ–induced PD-L1 expression via inhibition of JAK2/STAT1 signaling and decreases EGF-induced PD-L1 expression through inhibition of EGFR/Akt signaling	NSCLC cells and mouse melanoma cells	[103]
Hesperidin	↓PD-L1 expression	Suppresses Akt and NF-κB signaling and inhibits the activation of matrix metalloproteinases such as MMP-9 and MMP-2	TNBC cells	[104]
Quercetin	↓PD-L1 expression	Inhibits the IFN-γ dependent PD-L1 upregulation	Melanoma cells	[105]
Icaritin	Synergistic effect with anti-PD-1 Ab↓PD-L1 expression	Blocks IKK complex formation and NF-κB translocation which promote PD-L1 expression	Liver cancer cells	[106]
Baicalein	↓PD-L1 expression	Inhibits STAT3 and IFN-γ-induced PD-L1 expression	Liver cancer cells	[107]
Curcumin	↓PD-L1 expression	Inhibits STAT3 pathway	Tongue squamous cell carcinoma	[108]
	↓PD-L1 expression Synergistic effect with anti-CTLA-4 antibody	Inhibits CSN5 activity and reduces deubiquitination of PD-L1	Mouse breast cancer in vivo model	[109]
	Synergistic effect with anti-PD-L1 Ab	Increases CD8^+^ T cell stimulatory activity of DC by decreasing NF-κB and STAT3 signaling	Mouse colon cancer in vivo model	[110]
	Synergistic effect with anti-PD-L1 Ab	Inhibits NF-κB-induced cytokines secretion in cytotoxic T cells reducing Treg recruitment and promoting anti-tumor T cell infiltration	Mouse melanoma in vivo model	[111]
	↑CTLA-4 expression	N/A	Mouse spleen CD4+CD25+ Tregs	[112]
Bisdemethoxycurcumin	Synergistic effect with anti-PD-L1 Ab	Increases CD8^+^ T cell tumor infiltration and IFN-γ and granzyme B secretion	Mouse bladder cancer in vivo model	[113]
Resveratrol	↑PD-L1 dysfunction	Increases cytotoxic T cells sensitivity by *N*-linked glycosylation and dimerization of PD-L1 in a SIRT1-, AMPK-, and GSK3β-independent manner	Breast cancer cells	[114]
	↓PD-L1 expression	Inhibits thyroxine-induced PD-L1 expression	Oral cancer cells	[115]
	Synergistic effect with anti-PD-1 Ab	Induces immunogenic cell death by enhanced cell apoptosis and increased mature DCs and cytotoxic T cells	Mouse ovarian cancer in vivo model	[116]
	↑PD-L1 expression	Increases surface expression of PD-L1 by HDAC3/p300-mediated NF-κB signaling and decreases tumor cell survival via inducing apoptosis and DNA damage through the activation of caspase 3	Breast cancer cells and colorectal cancer cells	[117]
	↑CTLA-4 expression	N/A	Mouse spleen and thymus CD4^+^CD25^+^ Tregs	[118]
	↑CTLA-4 expression	N/A	Mouse peripheral blood Tregs	[119]
Polydatin	↓PD-L1 expression	Enhances miR-382 and inhibits miR-382-induced PD-L1 expression	Colorectal cancer cells	[120]
Caffeic Acid Phenethyl Ester (CAPE)	↓PD-L1 expression	Inhibits NF-κB pathway and regulates LMP1—induced PD—L1 expression	NPC cells	[121]
Gallic acid	↓PD-L1 expression	Suppresses EGF binding on EGFR resulting PI3K/AKT pathway inhibition, p53 and miR-34a upregulation and PD-L1 downregulation	NSCLC cells	[122]
Emodin	↓PD-L1 stabilization and PD-1 binding	Reduces TNF-α-induced PD-L1 stabilization and PD-1 binding	Breast cancer cells	[109]

**Table 2:** List of non-polyphenolic compounds and their effects with mechanisms on immune checkpoints modulation.

Group	Compound	Effect	Mechanism of Effect	Cell Type/Model	Refs
Terpene	Lycopene	Synergistic effect with anti-PD-1 Ab ↓PD-L1 expression	Reduces IFN-γ-induced PD-L1 expression by activating JAK2 signaling and AKT inhibition.	Mouse lung cancer in vivo model	[92]
	Fraxinellone	↓PD-L1 expression	Reduces STAT3 activation and HIF-1α protein synthesis via JAK/Src, mTOR/p70S6K/eIF4E and MAPK pathways	Lung cancer, cervical cancer, colon cancer and hepatic cancer cells Human lung cancer xenografted mouse model	[198]
	β-elemene	↓PD-L1 expression	Inhibits Akt activation and its dependent PD-L1 expression	Esophageal cancer cells	[199]
	Cryptotanshinone	Synergistic effect with anti-PD-L1 Ab	Develops long-term anti-tumor immunity and increased tumor infiltration of CD8+ T cell	HCC bearing mouse model	[200]
	Triptolide	↓PD-L1 expression	Reduces IFN-γ-induced PD-L1 expression	Breast cancer cells	[201]
		↓PD-L1 expression	Reduces IFN-γ-induced PD-L1 expression and reactivate CD4^+^ T cell	Glioma cells	[202]
		↓PD-L1 expression	Reduces IFN-γ-related JAK2-STAT1 pathway and decreases PD-L1 expression	Oral cancer cells	[203]
	Cannabinoid	↓PD-L1 expression	Reduces PD-L1 expression by downregulation of PAK1 and Kras activity	Human pancreatic cancer cells and mouse pancreatic cancer mouse model	[204]
Saponins (terpenes)	Ginsenoside Rg3	↓PD-L1 expression	Reduces cisplatin-induced PD-L1 expression by decreasing NF-κB p65 and Akt activity	NSCLC cells	[205]
	Ginsenoside Rg2	↓PD-L1 expression	Reduces cisplatin-induced PD-L1 expression by decreasing EGFR, PI3K and Akt activity	NSCLC cells	[206]
	Ginsenoside Rk1	↓PD-L1 expression	Reduces PD-L1 expression by inhibiting NF-κB and Bcl-2	NSCLC cells	[207]
	Panaxadiol	↓PD-L1 expression	Reduces PD-L1 expression by suppressing HIF-1α and STAT3	Colon cancer cells	[208]
	Platycodin D	↓PD-L1 expression	Reduces PD-L1 expression via extracellular release	Lung cancer cells	[209]
	Triterpenoid saponins (*Anemone flaccida*)	↓PD-1 and PD-L1 expression	Decreases the number of Tregs and increases T cells Induces apoptosis of HCC by blocking the activation of PD-1/PD-L1, ERK1/2, p38 MAPK, JNK and STAT3.	HCC bearing mouse model	[210]
	Diosgenin	Synergistic effect with anti-PD-1 Ab	Enhances T cell immune response via modulating intestinal microbiota and inducing T cell infiltration and IFN-γ secretion	Melanoma bearing mouse model	[211]
Isothiocyanate	Sulforaphane (SFN)	↓PD-L1 expression	Inhibits monocytes from MDSCs formation	Human GCM-treated CD14^+^ monocytes	[91]
Alkaloid	Camptothecin	↓PD-L1 expression	Reduces PD-L1 expression and upregulates the secretion of pro-tumorigenic cytokines	Colon cancer cells	[212]
		↑PD-L1 expression	Induces double-strand break-dependent PD-L1 expression via ATM/ATR/Chk1 activation and STAT1/3-IRF1 pathway	Osteosarcoma, lung cancer and prostate cancer cells	[213]
*Rhus verniciflua* Stokes extract	Eriodictyol, fisetin, liquiritigenin, quercetin	Obstructed PD-1/PD-L1 interaction	N/A	ELISA	[214]
*Anoectochilus formosanus* extract	extract	↓PD-L1 expression	Reduces PD-L1 expression and protein accumulation by its free radical scavenging capacity	Oral cancer cells	[215]
*Prunus mume* extract	MK615	↓PD-L1 expression	Reduces PD-L1 expression by inhibiting NF-κB	Melanoma bearing mouse model	[216]

## Data Availability

Not applicable.
